# Investigation of Body Development in Growing Holstein Heifers With Special Emphasis on Body Fat Development Using Bioelectrical Impedance Analysis

**DOI:** 10.3389/fvets.2021.724300

**Published:** 2021-08-20

**Authors:** Kathrin Heine, Viktoria Kichmann, Miriam K. von Kuhlberg, Ingrid Vervuert, Lisa Bachmann, Jens Lippmann, Jutta Gottschalk, Susanne Reitemeier, Ilka Steinhöfel, Almuth Einspanier

**Affiliations:** ^1^Albrecht-Daniel-Thaer-Institut e.V., Leipzig, Germany; ^2^Faculty of Veterinary Medicine, Institute of Physiological Chemistry, University of Leipzig, Leipzig, Germany; ^3^Faculty of Veterinary Medicine, Institute of Animal Nutrition, Nutritional Diseases and Dietetics, University of Leipzig, Leipzig, Germany; ^4^Leibniz Institute for Farm Animal Biology (Forschungsinstitut für Nutztierbiologie), Dummerstorf, Germany; ^5^Saxony State Office for Environment, Agriculture and Geology, Dresden, Germany

**Keywords:** heifer, body fat, bioelectrical impedance analysis, skeletal growth, body development, fat development

## Abstract

This study analyzed skeletal development, body condition, and total body fat development of growing heifers. A total of 144 female primiparous Holstein cattle from four commercial dairy farms with different degrees of stillbirth rates were examined during the rearing period. This included measurements in body condition, fat tissue, metabolic, and endocrine factors. Pelvic measurements and the sacrum height were analyzed to assess skeletal development. The body condition was classified via body condition scoring, bioelectrical impedance analysis (BIA), back fat thickness measurements, and the body mass. For the first time, BIA was used as an appropriate method to evaluate the fat tissue content of cattle throughout the rearing period. This analysis technique can be performed on heifers aged 8–15 months. Throughout that period, the fat content decreased while the skeletal development increased. In addition, high free fatty acid concentrations in serum of the animals with high frame development were found, supporting our hypothesis that stored energy of body fat deposits is used for skeletal growth. Furthermore, we were able to demonstrate complex endocrine relationships between fat metabolism and skeletal growth by using specific markers, such as leptin, insulin growth factor-1 (IGF-1), and estradiol (E2). Food analysis showed high crude protein (CP) levels in the total mixed ration above recommendation for daily protein intake of all farms. However, there was a positive correlation between CP and the body frame measurements in our study. In summary, we established a novel regression formula for BIA analysis (“*BIA-Heine*”) in heifers to evaluate the body composition throughout different ages and physiological stages in the development of heifers. This special formula allows the evaluation of fat tissue without a whole-body analysis and therefore provides an innovative technique for animal welfare support.

## Introduction

As a result of excessive body condition with a consequent obstruction of the birth tract by intrapelvic fat depots, increased stillbirth rates are reported in primiparous cattle (1–3). One critical point for excessive body condition during the rearing period of cattle is the age of puberty (4). Therefore, young cattle rearing should focus on continuous growth with the development of a large body frame and low body fat content. It has been shown that compensatory growth is associated with increased body fat (5), which accumulates throughout further rearing (2, 6). It is often recommended [e.g., (7)] to determine regularly the body mass (BM) for an optimal rearing process. However, this parameter on its own does not provide any information about the whole-body composition, especially concerning body fat content or skeletal mass (8). Therefore, the combination of frame formation assessment and body condition is expected to be more useful.

The frame development is constructed on the measurement of sacrum height (SH) as well as the pelvic diameter (8, 9). The body condition is calculated by body condition scores (BCS) (10) and back fat thickness (BFT) (11), while there is a close correlation between BFT and total body fat content. This close correlation is described in several studies using the invasive “gold standard” of whole-body analysis to examine the components fat, protein, and water by slaughtering (12).

Unfortunately, there are hardly any reliable studies about body condition and fat development of rearing heifers. So far, it is well-known that after the onset of puberty, the changes in metabolic and hormonal situation lead to an increase in fat accumulation related to the cow breed (13). Furthermore, fat deposits are dominating depending on the breed differences, e.g., beef breed have more subcutaneous whereas milk breeds have more visceral fat deposits (14). However, to analyze the development of body fat tissue irrespective of age and breed, novel non-invasive and repeatable methods are required to obtain the individual body components on alive animals to support animal welfare. The bioelectric impedance analysis (BIA), established for human body composition measurement, is a measurement of the total electrical resistance of the body (= impedance) across the volume (12). In veterinary medicine, it has been rarely applied, such as to evaluate carcasses of beef cattle, rabbits, and sheep (15–18). In previous studies, the total body fat content of lactating cattle was compared with BIA (12, 19), but this method was never used for rearing cattle.

Therefore, the objectives of our study were to determine the course of body development, especially total body fat content, of growing Holstein heifers at age of 8–15 months using the non-invasive technique BIA, and to classify it in combination with the evaluation of feeding, endocrinology, and blood chemistry in order to avoid excessive fat accumulation during the rearing period to reduce later calf losses.

## Materials and Methods

### Study Design

This study was part of a project to analyze stillbirths in Holstein heifers. In this part of the project, we analyzed the body development of rearing heifers on dairy farms with various stillborn rates to characterize the causative differences during the rearing on these farms.

All experimental procedures were performed in accordance with the German Animal Welfare Act and approved by the federal animal experimentation authorities (Landesdirektion Leipzig, file number 48/18).

The study was conducted on four commercial dairy farms (farms A–D) in Saxony from March 2019 to April 2020. Animal numbers on the farms varied from 407 to 1,001 cattle with different degrees of stillbirths (Table 1). The farms were selected for their stillborn rates in the 3-year history. Calf losses in this study were defined as stillborn or died within the first 3 days of life.

**Table 1:** Overview of parameters of the four dairy farms (A–D) from 2017 to 2020.

**Farm**		**A**	**B**	**C**	**D**
Herd of cow	Numbers	485	1,001	751	407
Calf losses (2017–2019)	%	10.3	7.6	10.8	5.3
Calf losses from heifers (2019–2020)	%	12.0	3.0	14.0	9.0
Number of study animals	Numbers	37	37	36	34

For each farm, four examinations were conducted throughout the year, and animals were examined for skeletal growth and body condition. For each examination, 8–10 animals were randomly selected on each farm. Measurements from each examination were analyzed together. A total of 144 Holstein cattle aged 8–15 months were examined at all four farms. Total body fat content was determined using the BIA technique as described for different cattle breeds (12, 19), combined with measurements for skeletal growth, endocrine, and blood chemistry parameters. In addition, food samples were taken to obtain information on the influence of ration formulation on fat and body development.

### Measurements

#### Bioelectric Impedance Analysis

The principle of BIA is based on measuring the total electrical resistance of the body to calculate the total body fat content excluding individual local fat depots. In this study, the impedance meter Nutriguard MS2 (Data Input GmbH, Wedemark, Germany; measuring range, 5–100 kHz) was used with an impedance measuring point at 50 kHz as published elsewhere (12). The corresponding skin areas were trimmed, shaved, and cleaned, and the adhesive electrodes (BIANOSTIC AT®, Data Input GmbH, Wedemark, Germany) were applied to these prepared body positions. The cranial electricity electrode was placed 2 cm caudal of the shoulder blade bone (spina scapulae), and the sensor electrode ~10 cm caudal of the electricity electrode. In the caudal area, the electrode pair was placed at the same height as the cranial electrode pair, and the position 10 cm in front of the caudal electricity electrode was chosen for the sensor electrode (12). The BIA measurement was carried out three times per investigation at each animal to calculate an average value from the individual measurements. The distances between the sensor electrodes (MD) were measured and recorded with a tape measure.

#### BCS

Overall, the BCS is an established and frequently used method in the literature for assessing the body condition of cattle (10, 20). Our BCS followed these recommendations by obtaining BCS through visual and palpatory inspection of the animals according to the scoring table (10). The published scoring scale of 1–5 in 0.25 steps was used, starting from 1 for cachectic to 5 for obese animals.

#### BFT

The BFT is an objective measurement obtained by ultrasound using a linear transducer (Esaote Tringa Linear, ESAOTE Biomedica Deutschland GmbH, 50858 Cologne, Germany) according to the published measurement point (11, 20). The transducer was placed on the right side between the coxae and the ischiadic tube. The BFT was determined in centimeters using the measurement tool of the ultrasound device at 5 MHz.

#### BM and Animal Data

To determine the BM, each animal was separated on a flat surface using panel grids, and the weight was determined by a mobile animal scale with digital display (0–1,500 kg, True Test Patura, PATURA KG, Laudenbach, Germany).

In addition, the herd management programme “Herde” (dsp-Agrosoft GmbH, 14669 Ketzin/Havel, Germany) or “AGRACOM Superkuh” (CLAAS KGaA mbH, 33428 Harsewinkel, Germany) was used to record the data about parentage and date of birth of each heifer.

#### SH

The SH was used to estimate the frame size (9) and was determined with a metallic measuring rod (Hauptner Herberholz GmbH & Co. KG, Solingen, Germany) as well as a movable measuring arm. The back line was examined by a transverse line in front of the two hips (21, 22).

#### Pelvic Dimensions

The measurement of the pelvic dimensions (8, 9) is an important assessment of the skeleton. As described by Essmeyer (4), it includes the distances of the ischial tuberosities (ischial width) and the hip tuberosities (pelvic width) of the heifers. For the pelvic width, the distance of the hip humps was analyzed such as the distance between the lateral iliac angles. A pelvic circle (Johannes Hammer, wissenschaftliche Apparate, Leipzig, Germany) was used for the measurement of the pelvis, allowing direct measurement calculation.

#### Feed Analysis

Assessing energy and nutrient supply of the animals will allow conclusions about their growth and body condition. Therefore, one sample per examination of the total mixed rations (TMR) was taken. After the analysis, the results have been averaged.

The dry matter (DM) was determined after oven drying (103°C) to constant mass, and samples were ground to 1 mm in size. The crude protein (CP**)** was determined by Weende analysis (23).

For the calculation of energy, equations from the Society for Nutrition Physiology and Committee for Needs Standards (2001/2004) were used (24). TMR was analyzed by using the “Penn State Particle Separator” for the assessment of the physically effective neutral detergent fibre (NDF)AQQ21 [peNDF; (25)].

#### Blood Sample Analysis

##### Chemical Analysis

One blood sample per animal was taken from the V. caudalis mediana (serum gel tubes, Sarstedt AG & Co. KG, 51588 Nümbrecht, Germany). After clotting (30 min), the samples were centrifuged with the SERVOspin next benchtop centrifuge (servoprax GmbH, 46485 Wesel, Germany) at 4,000 rpm for 10 min; serum was separated and then stored at −20°C until analysis.

The samples were analyzed for blood chemistry, especially for urea (BUN), ß-hydroxy butyrate (BHB), glutamate dehydrogenase (GLDH), and gamma-glutamyl transferase (GGT) using the non-accredited method FMUAA 172 2019-03 (bichromatic measurement) by the LKS–Landwirtschaftliche Kommunikations- und Service GmbH (Lichtenwalde, Germany).

The analysis of free fatty acids (FFA) was carried out to obtain information about metabolic situation using the NEFA test kit from Randox Laboratories Ltd. (Crumlin, UK).

##### Endocrine Measurements

To identify interactions between growth rate and lipid metabolism in relation to BIA, endocrine measurements were performed. In addition, the number of prepubertal or post-pubertal animals was determined by progesterone blood concentration.

Two different sex steroids, progesterone (P4) and estradiol (E2), were analyzed by enzyme immunoassay (EIA) modified according to Gottschalk (26) to determine the pubertal stage of the animals (P4) and to identify details on the fat metabolism (E2). Insulin growth hormone [insulin growth factor-1 (IGF-1)] was analyzed using a commercial EIA (IGF-1 ELISA, IBL International GmbH sales via Tecan Group AG, Männedorf, Switzerland) as one of the most important hormones on skeletal growth. For fat metabolism, the hormones insulin and leptin were determined by radioimmunoassay [RIA; Multi-Species Leptin RIA, BIOTREND Chemikalien GmbH, Cologne, Germany; for insulin according to Gottschalk et al. (27)].

## Statistical Analysis

Data were prepared using Microsoft Excel (Microsoft Corporation, Redmond, Washington, USA). The IBM SPSS Statistics 27 for Windows program (IBM Deutschland GmbH, 71139 Ehningen, Germany) was used for statistical analysis. The results of the four examinations per farm were summarized to exclude seasonal variation.

All data were checked for normal distribution using the Kolmogorov–Smirnov test, and for significant differences between the farms using multivariate analysis of variance (mANOVA) with a subsequent *post-hoc* test by Bonferroni or Kruskal/Wallis test per Dunn–Bonferroni. The significance level was *p* ≤ 0.05.

In order to detect relations between different parameters, the correlation coefficient according to Pearson was determined.

For the descriptive evaluation of the data, mean value, and standard deviation were obtained.

### Calculation of Regression Formula for the Evaluation of the BIA in Dairy Heifers

A regression formula from adult dairy Fleckvieh cattle was published using measurements with 5 and 50 kHz (12). We adapted it to the situation in growing Holstein heifers for their total body fat content analysis. Therefore, our regression formula had to be modified to calculate the total fat content of Holstein heifers by using only 50-kHz measurements. The following adapted regression formula, called “*BIA Heine*,” was used to evaluate the BIA measurement in Holstein heifers:

$$\begin{array}{l} \text{Fat }(\text{kg})\;=\;117.447\;-\;0.193\;(\text{SH }(\text{cm})\;\times \text{ MD }(\text{cm})\text{/R50 }(\Omega ))\\ \;+0.000331\;{\text{BM}}^{2}\;(\text{kg}) \end{array}$$

SH, height of sacrum; MD, measuring distance between the sensor electrodes; R50, resistance frequency 50 kHz.

The adapted formula yields a corrected *R*^2^ of 0.89 with an RMSE of 17.08 kg and a deviation of 0.027 at the corrected coefficient of determination (*R*^2^) and 3.1 kg of the RMSE (root-mean-square error) from the original formula of Schneider (12).

## Results

### BIA

The analysis of the total body fat mass by using formula “*BIA Heine*” showed no significant differences (*p* ≥ 0.05) between the four farms (Table 2). However, farm D had the highest recorded body fat content (63.86 ± 10.73 kg), and farm A the lowest (59.10 ± 10.08 kg). Farm B had a body fat content of 59.59 ± 13.49 kg, and farm C had a higher body fat content of 62.52 ± 10.35 kg.

**Table 2:** Overview of the measured parameters of the study sorted by the farms (A–D).

	**Farm**	**A**		**B**		**C**		**D**	
	***N***	**37**		**37**		**36**		**34**	
**Parameter**		**Mean**	**Std.-dev**.	**Mean**	**Std.-dev**.	**Mean**	**Std.-dev**.	**Mean**	**Std.-dev**.
BIA	Fat “*BIA Heine*” (kg)	59.10	10.07	59.59	13.49	62.52	10.35	63.86	10.73
	Fat “*BIA Heine*” (%)	16.82	3.18	16.03	3.95	17.98	3.12	19.29	4.23
	Fat “*cow*” (kg)	67.49	8.49	72.26	8.02	70.02	8.29	72.17	12.16
	Fat “*cow*” (%)	19.10	1.72	19.43	2.94	20.12	2.26	21.39	1.87
	MD (cm)	119.65	7.49	127.49	5.92	120.19	6.40	122.21	10.14
	MA (m^2^)	1.38	0.11	1.51	0.13	1.38	0.10	1.41	0.17
Skeletal development	SH (cm)	133.51	5.01	136.41	6.25	133.81	5.08	132.74	6.11
	Pelvic width (cm)	41.22	2.48	45.00	1.93	41.56	2.59	40.15	2.56
	Ischial width (cm)	14.81	1.70	16.16	1.12	15.78	1.66	15.00	1.81
Animal data	Age (months)	12.16	1.38	13.17	1.34	11.26	1.49	11.17	1.30
	BM (kg)	355.27	47.60	377.35	51.56	350.50	42.19	338.68	56.46
Body condition	BFT (cm)	0.85	0.18	0.95	0.17	0.91	0.17	0.95	0.25
	BCS	3.57	0.25	3.54	0.18	3.52	0.24	3.65	0.17
Blood chemistry	BUN (mmol/L)	3.25	0.84	2.86	0.96	3.64	1.42	3.46	0.98
	FFA (μmol/L)	126.62	58.99	137.49	74.91	132.11	96.79	72.32	44.67
	BHB (μmol/L)	383.77	116.45	447.04	129.03	499.06	116.78	421.24	228.18
	GLDH (U/L)	26.56	26.57	16.33	17.45	19.60	10.95	18.99	7.48
	GGT (U/L)	18.32	4.58	19.57	4.85	19.19	3.57	20.79	7.19
Endocrine analysis	P4 (ng/ml)	5.96	7.88	6.99	9.64	5.40	7.52	5.82	14.46
	E2 (pg/ml)	22.68	6.32	18.02	7.62	21.72	6.24	20.67	5.82
	Leptin (ng/ml)	12.11	7.36	12.27	4.65	11.60	7.56	10.07	4.16
	IGF-1 (ng/ml)	368.07	72.30	285.77	116.72	321.36	99.83	346.44	95.39
	Insulin (nmol/L)	0.20	0.12	0.23	0.11	0.23	0.17	0.33	0.16

*Mean value and standard deviation (Std.-dev.) are given for each parameter*.

The results obtained by the formula “*BIA Heine*” were than compared with the formula for whole-body fat analysis for cows by Klawuhn and Staufenbiel (11), called “*cow*” [fat (kg) = 4.77 (BFT (mm) +26.8)]. The “*cow*” analysis resulted into a regression formula based on back fat measurement: changes of millimeter back fat related to general body fat of 5.89 kg per millimeter.

By using “*cow*,” farm A showed the lowest body fat content of 67.49 ± 8.50 kg, whereas the highest body fat content with 72.26 ± 8.02 kg was obtained by farm B followed by farm C (70.02 ± 8.29 kg) and farm D (72.17 ± 12.16 kg) (Table 2).

Comparing both formulas in farm A, the results of “*cow*” differed 1.14% from the results of the “*BIA Heine*” analysis. In farm B, we detected a variation of 1.21%, in farm C 1.12%, and 1.13% for farm D between “*BIA Heine*” and “*cow*.”

**Figure 1:**
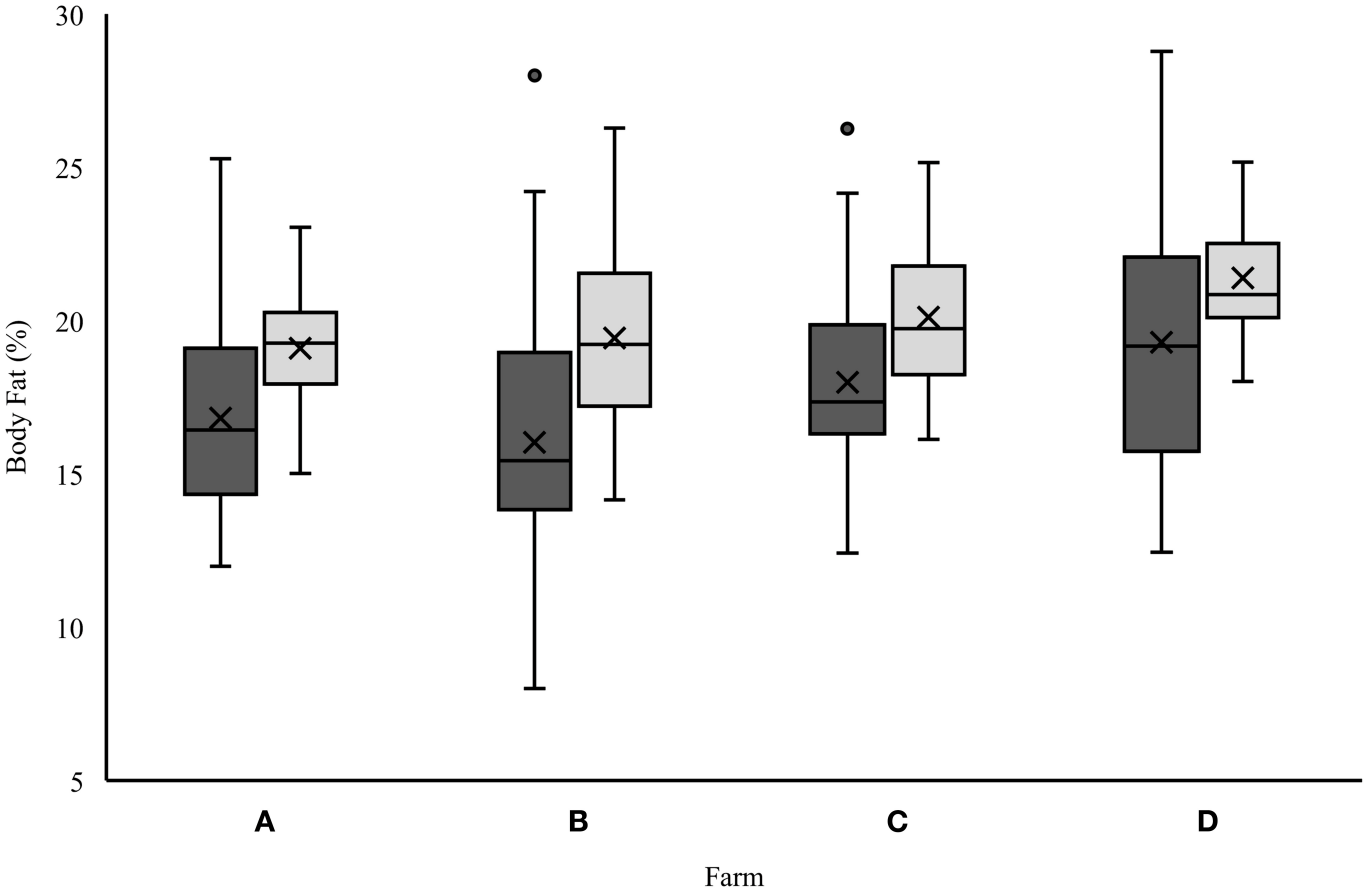
Comparative analysis of percentage fat content of “*BIA Heine*” (dark gray boxes) and “*cow*” (light gray boxes) at the four farms (A–D).

In order to assess the body fat content depending on the BM, the percentage of fat was calculated for both formulas (Figure 1). Farm D was detected to have the highest value with 19.29 ± 4.23% (“*BIA Heine*”) or rather 21.39 ± 1.87% (“*cow*”). The lowest body fat percentage was found for farm B (“*BIA Heine*”: 16.03 ± 3.95%) and for farm A (“*cow*”: 19.10 ± 1.72%). For “*BIA Heine*,” farms A (16.82 ± 3.18%) and C (17.98 ± 3.12%) ranked in the middle, whereas farms B and C did for “*cow*.” Significant differences with *p* ≤ 0.001 were detected between farms B and D (“*BIA Heine*” and “*cow*”) and between farms A and D (“*cow*” only). There was only a difference of 1.15% to the values of “*BIA Heine*,” which was not significant (Figure 1).

**Figure 2:**
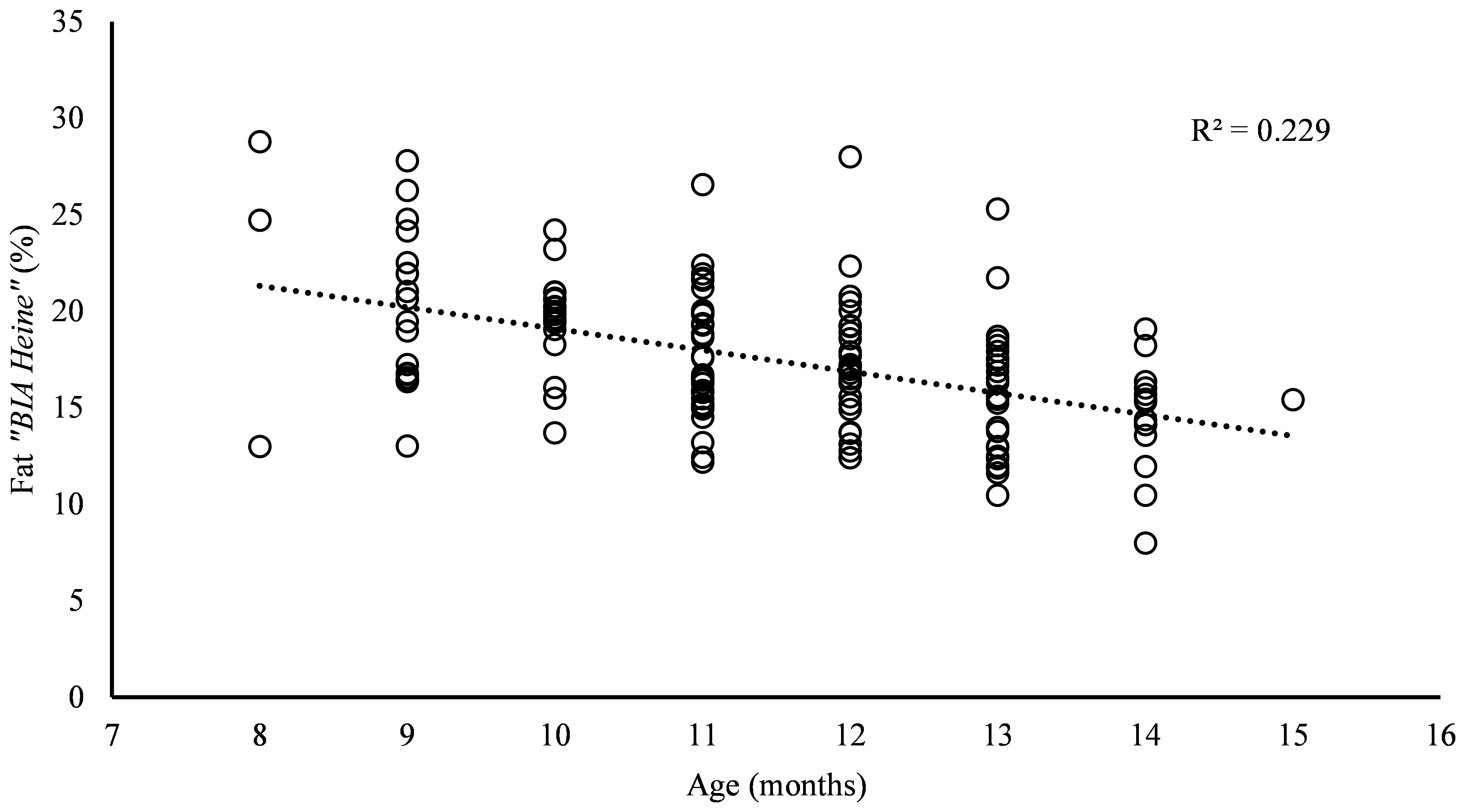
Relation between the percentage fat content of “*BIA Heine*” ° and the age. All study animals of farms A–D are represented. The coefficient of determination (*R*^2^) is given.

Furthermore, there is a negative correlation between percentage body fat and age (−0.48; *p* ≤ 0.01), with *R*^2^ of 0.23 (Figure 2).

The body volume was obtained by an indirect analysis of the measuring area (MA) through the height of the sacrum and the distance between the sensor electrodes. The MA was largest in farm B at 1.51 ± 0.13 m^2^, followed by farm D at 1.41 ± 0.17 m^2^, which was a significant (*p* = 0.02) difference from farm B. Both farms A and C had identical MAs of 1.38 m^2^, which were significantly (*p* ≤ 0.002) lower than that of farm B, but not farm D (*p* = 0.3; Table 2).

Among the measured BIA values, farm B had the longest measuring distances (MD) of the electricity electrodes (127.49 ± 5.92 cm) and sensor electrodes (110.27 ± 6.77 cm). The distance between the sensor electrodes covered almost the entire torso length, confirming that animals from farm B had the longest torso length. Animals with the shortest torso length were represented by farm C (103.24 ± 5.77 cm) with a significant difference to farm B (*p* = 0.018). The second largest animals were in farm D with 106.24 ± 9.22 cm, followed by farm A with 103.78 ± 6.25 cm, but they did not significantly differ (*p* ≥ 0.05; Table 2).

**Figure 3:**
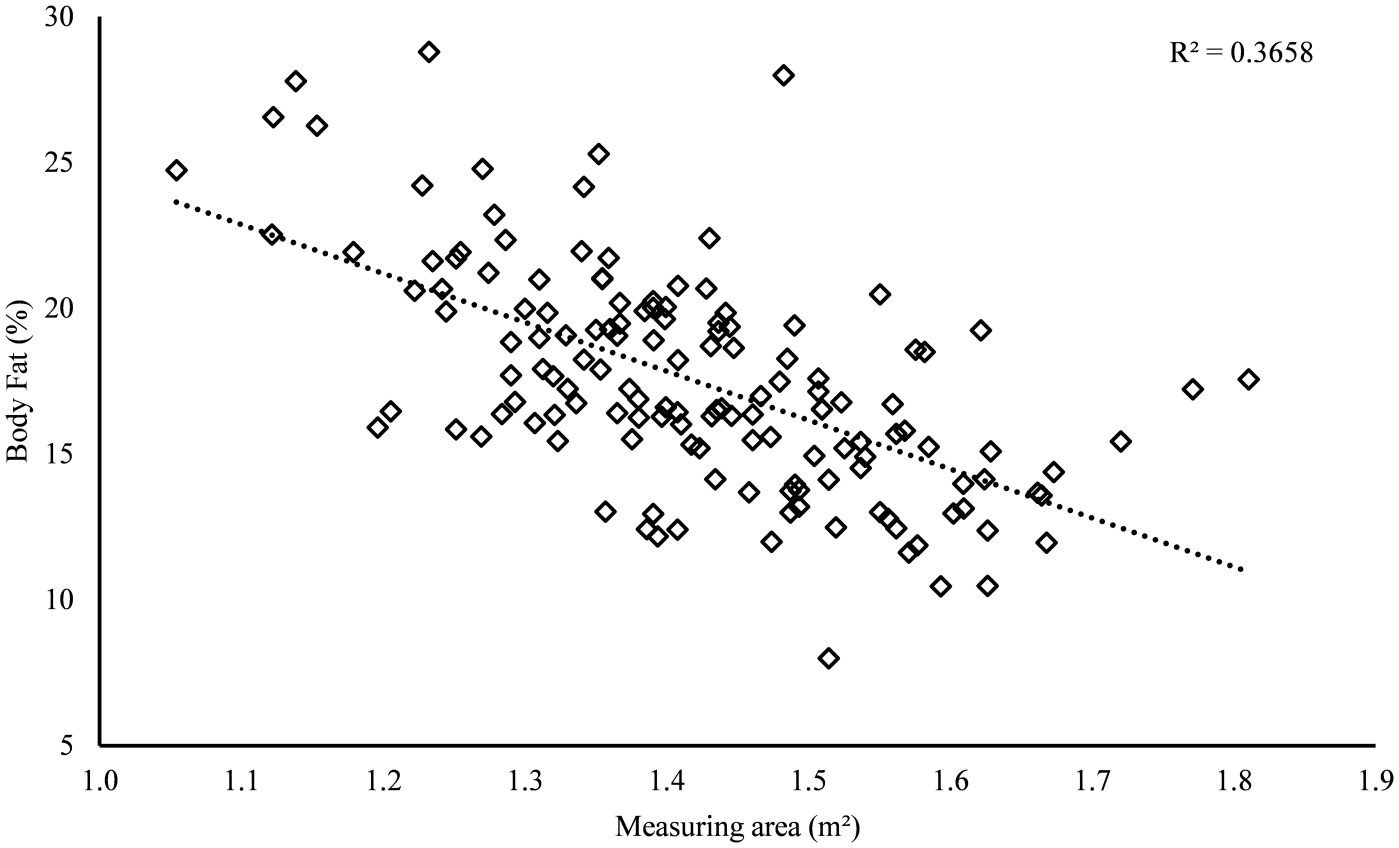
Relation between the percentage fat content of “*BIA Heine*” and the measurement area. All study animals of farms A–D are represented. ♢The symbol means: percentage fat content of “*BIA Heine*”.

Taking all four farms together, the further growth of the heifers could be confirmed over age by a positive correlation between age and the MD (0.42; *p* ≤ 0.01), as well as age and the MA (0.57; *p* ≤ 0.01). However, there is a negative correlation between MA and percentage of body fat (−0.61; *p* ≤ 0.01; see Figure 3). The relationship between MA and age showed a coefficient of determination (*R*^2^) of 0.32.

### Skeletal Development, Body Condition, and Parentage Data

Analyzing SH as a parameter for skeletal development, the tallest animals were present in farm B (136.40 ± 6.25 cm), and the shortest in farm D (132.74 ± 6.11 cm), with no significant difference (*p* = 0.06) between all four farms (Table 2). However, there was a strong negative correlation (−0.63; *p* ≤ 0.01) between SH and percentage of body fat content. These results were in line with our pelvic width data, which were significantly larger in farm B with 45.00 ± 1.93 cm compared to the other three farms (*p* ≤ 0.001). Furthermore, there was a difference in ischial width between farm B (16.16 ± 1.12 cm) and farm D (15.00 ± 1.81 cm; *p* = 0.04; Table 2). There was also a significant difference between farm B and farm A (14.81 ± 1.70 cm; *p* = 0.002), having the smallest ischial width (Table 2). For all farms, both ischial and pelvic width were negatively correlated (*p* ≤ 0.05) with the percentage of fat content.

Similar to the results of SH, the BM correlated negatively (−0.54; *p* ≤ 0.01) with the percentage of fat content. Because of BM analysis, farm B had the heaviest animals (377.35 ± 51.56 kg), with a significant difference to the other farms (*p* ≤ 0.03), whereas the animals from farm D displayed the lowest BM (338.68 ± 56.46 kg). BM and age correlated positively (*p* ≤ 0.01) with frame development and body condition.

For both measurements of body condition (BFT and BCS), no difference (*p* > 0.06) between the four farms has been detected. However, farm A had the lowest (0.85 ± 0.18 cm) and farm B the highest (0.95 ± 0.17 cm) BFT (Table 2). BFT correlated positively with total fat mass (0.28; *p* ≤ 0.01) and negatively with the percentage of fat (−0.22; *p* ≤ 0.01).

Farm D had the highest BCS (3.65 ± 0.17) and farm C the lowest (3.52 ± 0.24) (Table 2). BCS also correlated positively with total body fat mass (0.18; *p* ≤ 0.03).

**Figure 4:**
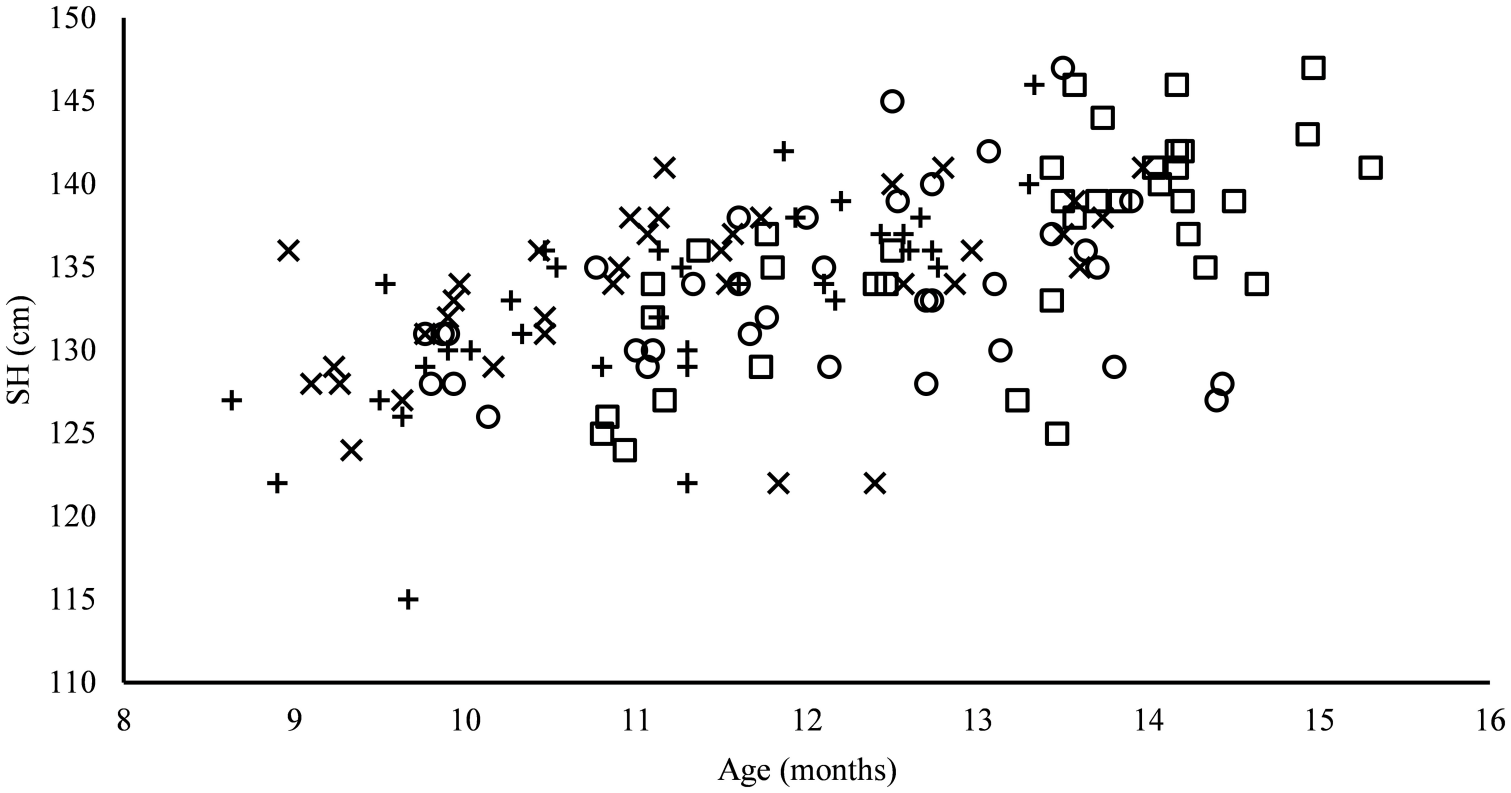
Relation between SH and age across all study animals of farms A–D. Farm A °, farm B □, farm C x, and farm D +.

The heifers of farm B were the oldest animals in this study (13.17 ± 1.34 months), with a significant difference to farms C (11.26 ± 1.49 months; *p* ≤ 0.001) and D (11.17 ± 1.30 months; *p* ≤ 0.001; Table 2). The age was significantly positive correlated with the parameters of frame development (SH: 0.58, MA: 0.57, pelvis width: 0.68, and ischial width: 0.47; *p* ≤ 0.01) and body condition (0.29; *p* ≤ 0.01) in all farms. Looking at the skeletal development throughout the experimental period, there was an age-dependent increase in frame measure (Figure 4) with a relationship between hip width and age (*R*^2^ = 0.48).

Parentage data of study animals showed different genetics between the four farms. In farm C, there were 100% Holstein genetic animals. With 2.7% dual purpose breeds, farm B displayed a low genetic pairing with other breeds compared to farm A with 16.2%. The most diverse genetic parentage data observed in farm D with 23.5% (17.7% dual purpose breeds parentage genetic and 5.8% beef cattle genetic).

### Feed Analysis

Table 3 summarizes the analyzed CP, metabolized energy (ME), peNDF, and calculated intake per animal per day based on a mean DMI for 300–400 kg cattle according to Kamphues et al. (28).

**Table 3:** Analytical values of protein, structure, and energy in the TMR samples, and calculation of the supply situation at the four farms using the average DM intake of 6.2–7.8 kg per animal per day (28) compared to the literature recommendation.

**Parameter per kg DM**	**Farm**	***N***	**Mean**	**Std.-deviation**	**Calculated quantity taken in/animal/day**	**Recommendation according to Kamphues et al. (28)**
CP (g)	A	29	140.43	28.66	870–1,095	670–855 CP g/animal/day
	B	28	151.96	12.49	942–1,185	
	C	26	129.72	8.97	804–1,011	
	D	26	142.06	3.66	880–1,108	
ME (MJ)	A	29	9.27	0.82	57.5–72.3	59–75 ME MJ/animal/day
	B	28	9.78	0.40	60.6–76.3	
	C	26	8.83	0.23	54.8–68.9	
	D	26	9.31	1.17	57.7–72.7	
peNDF (%)	A	29	21.33	3.16	21.33	≥18–20%
	B	28	21.20	0.80	21.20	
	C	26	18.75	2.10	18.75	
	D	26	20.83	6.15	20.83	

The amount of CP was highest in farm B (151.96 ± 12.49 g/kg DM) and lowest in farm C (129.72 ± 8.97 g/kg DM).

In terms of ME, farm C had the lowest energy supply (8.83 ± 0.23 MJ/kg DM), whereas farm B had the highest value (9.78 ± 0.40 MJ/kg DM).

The analysis of peNDF for farms A (21.33 ± 3.16%) and B (21.20 ± 0.80%) was slightly above the recommendation of 18–20% (28). The lowest peNDF value was found in farm C (18.75 ± 2.10%).

The frame development of the heifers showed a highly significant positive correlation with CP and ME (*p* ≤ 0.01). BM correlates positively with CP (0.17, *p* ≤ 0.05) and ME (0.26, *p* ≤ 0.01). Body fat mass and the percentage of fat content are negatively correlated with CP and ME (*p* ≤ 0.01). ME correlated significantly positive with CP (0.43; *p* ≤ 0.01).

### Blood Chemical Analysis

Serum was used for blood chemical analysis. For BUN, there were no significant differences (*p* = 0.1) between the four farms (Table 2).

BHB was highest at farm C (499.06 ± 116.79 μmol/L), with significance vs. farms D (*p* = 0.002; 421.24 ± 228.18 μmol/L) and A (*p* = 0.001; 383.77 ± 116.45 μmol/L) (Table 2). Compared with food analysis, farm C showed the lowest content of ME in its rations with the highest blood BHB. On the other hand, farm A with low level of energy in their food ratio had the lowest level of BHB.

Looking at the liver specific enzyme GLDH, farm A showed the highest value (26.56 ± 26.57 U/L) with significant difference to farm B (*p* ≤ 0.001; 16.33 ± 17.45 U/L) (Table 2). There was also a significant difference between farm B and farm D (*p* = 0.04; 18.99 ± 7.48 U/L). GLDH showed a significant negative correlation (*p* = 0.02; −0.188) with fat kilogram “*cow*,” whereas the negative correlation (−0.089) with body fat kg "*BIA Heine*” was not significant (*p* = 0.3). Additionally, we found a coherence between GLDH and some hormones (see below).

For GGT, there were no significant differences (*p* = 0.12) between the four farms (Table 2).

**Figure 5:**
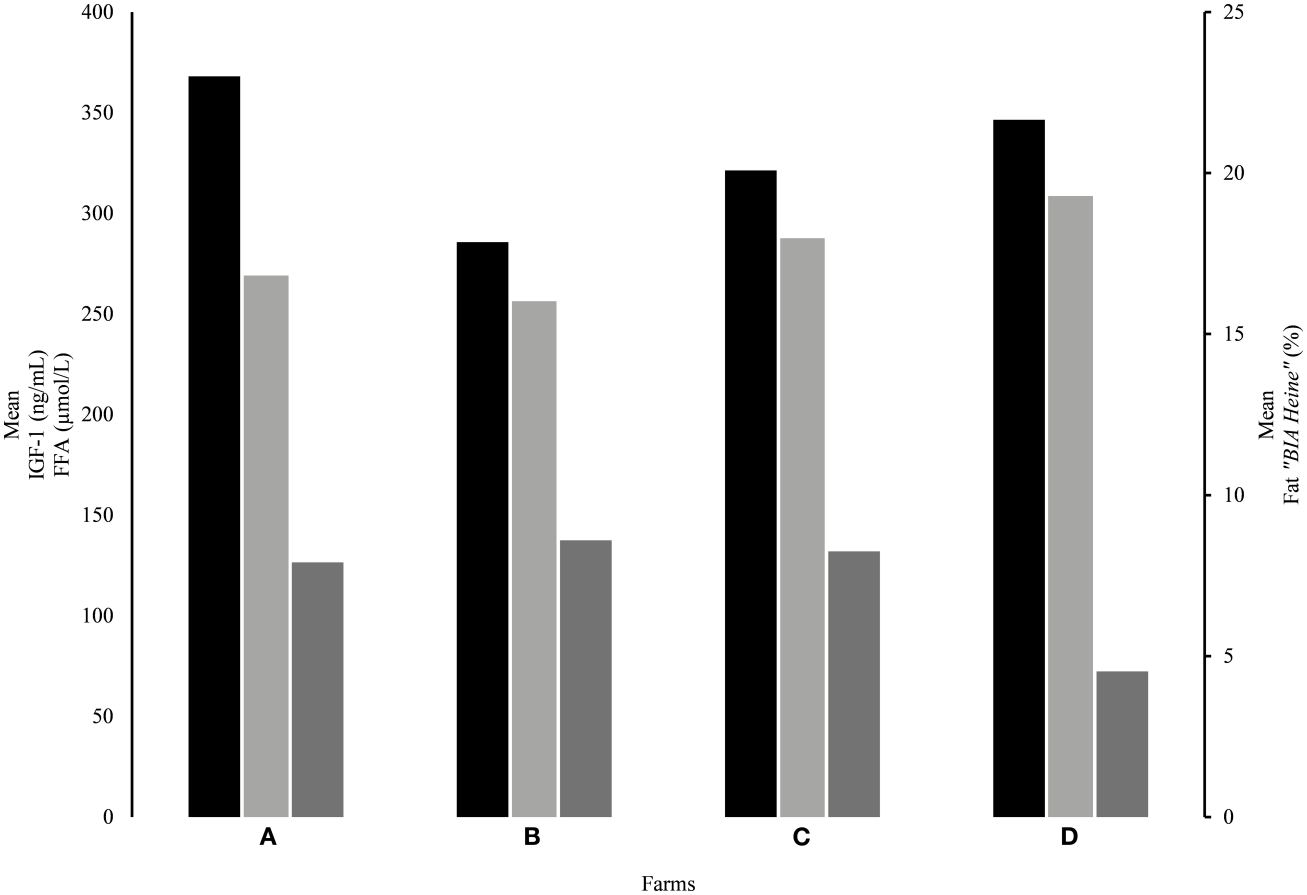
Relation between the mean values of IGF-1 (black column), FFA (dark gray column), and the percentage fat content of “*BIA Heine*” (light gray column) at the four farms (A–D). The fat content of “*BIA Heine*” (light gray column) is shown on the right y-axis. The FFA and IGF-1 are shown on the left y-axis.

Looking at FFA, farm D differed significantly (*p* ≤ 0.002) with the other farms, having the lowest value with 72.32 ± 44.67 μmol/L FFA, while farm B displayed the highest with 137.49 ± 74.91 μmol/L FFA (Table 2 and Figure 5). Compared to the FFA references (29), 27% of the study animals of farms A, B, and C showed elevated FFA. In contrast, this was observed in only 6% of the individuals of farm D.

### Endocrine Analysis

E2 content was lowest at farm B (18.02 ± 7.62 pg/ml) and significant vs. farm A (*p* = 0.001) with highest levels (22.68 ± 6.32 pg/ml), followed by farm C (21.72 ± 6.2432 pg/ml, *p* = 0.009; Table 3).

Serum IGF-1 showed significant differences (*p* = 0.001) between farm A (368.06 ± 72.30 ng/ml) and farm B (285.77 ± 116.72 ng/ml) (Table 2 and Figure 5).

Farm D had the highest insulin value (0.33 ± 0.16 nmol/L) with significant difference to the other farms (*p* ≤ 0.05). Farm A showed the lowest values for insulin (0.20 ± 0.12 nmol/L), whereas farms B and C showed identical mean insulin values of 0.23 nmol/L (SD: farm B: ±0.02 nmol/L; farm C: ±0.03 nmol/L) (Table 2). Serum insulin correlated positively with BFT (0.22; *p* ≤ 0.01), BCS (0.18: *p* ≤ 0.05), MD (0.21: *p* ≤ 0.01), MA (0.18; *p* ≤ 0.05), CP (0.25; *p* ≤ 0.01), and BHB (0.27; *p* ≤ 0.05) in all farms.

There was no significant difference between the serum leptin values of all four farms, ranging from 10.07 to 12.27 ng/ml (Table 2).

Furthermore, we found a coherence between GLDH and IGF-1 and E2: farm A had the highest GLDH values and at the same time the highest values for IGF-1 and E2. In contrast, farm B had the lowest GLDH with the lowest values for IGF-1 and E2.

### The Influence of Puberty

No statistical differences for the P4 levels (*p* = 0.1) were found between the four farms, ranging from farm C with the lowest P4 level (5.40 ± 7.52 ng/ml) to farm B with the highest level (6.99 ± 9.65 ng/ml) (Table 2). According to Day et al. (30), with values below 2 ng/ml described as prepubertal and values above 2 ng/ml as pubertal, our study animals were staged prepubertal for 17.5% of farm A, 20% of farm B, 25% of farm C, and 37.5% of farm D. Therefore, the influence on puberty is not statistically different between the farms.

## Discussion

This study analyzed skeletal development, body condition, and total body fat development of growing heifers in four commercial dairy farms with various stillborn rates to detect causative differences in the rearing period.

Postpubertal body fat accumulation resulting in intrapelvic obstruction is often discussed as a cause of dystocia in primipara (28). Therefore, the fat content of rearing animals in farms with different stillbirth rates was firstly obtained by BIA, which is a well-established method for the determination of human body composition (12). To examine the body composition of cattle over the rearing period, we compared the BIA measurements with body fat metabolism and skeletal growth by additionally taking food analysis, endocrine, and blood chemical parameters into account.

The food analysis confirmed that all TMR samples from the study farms fulfilled the recommendations for 8- to 10-month-old heifers (31) and adult cattle (28). In this study, the exact DMI could not be measured, but the mean DMI for 300–400 kg cattle with 6.2–7.8 kg per day per animal is postulated (28). Therefore, the postulated DMI was used to calculate the supply situation of the animals, but several factors still influence the DMI, such as feed quality, feed availability, water intake, and animal health (28).

With regard to CP in the TMR, all farms have been oversupplied. However, there was a significant positive correlation of CP content with BM, with skeletal measurements, and with BFT, as formerly published (32). At the same time, CP correlated significantly negative with the percentage of fat content. For example, farm B had the highest protein supply with the highest BM, the highest skeletal values, and the lowest body fat content.

In all farms, especially in farm B, some individuals had a higher BM (>400 kg) as expected under the recommended feeding. The higher BM and the high influence of CP on skeletal and body fat development indicate a better food conversion than previously expected. This could be based on genetic changes and the high growth potential of Holstein cattle (33).

To minimize excessive BM, current TMR composition should be optimized with special attention to protein supply and consideration of the high growth potential of the animals.

BM correlated also with ME; however, there was a difference in the energy supply between the farms. In farm C, the ME was lower than in the other farms. In addition, farm C had the lowest peNDF value. We could not find a detailed explanation for this context, but possible influences could be the composition and the quality of the TMR. Arguably, DMI in farm C was lower than estimated (due to TMR composition or quality), resulting in negative energy balance, as indicated by increased serum BHB levels (34). On the other hand, farm A had the second lowest level of ME in the study but the lowest serum BHB levels. This indicates an individual need of the animals. Although the mean values of BHB concentrations in both farms (C and A) are within the reference values (29), it should be taken into account that only few studies exist on BHB concentrations in growing heifers.

Therefore, in growing heifers, an optimal feed quality should be ensured to guarantee the maximum DMI and to avoid a negative energy balance.

The animals of farm C also had the highest calf losses during the study (see Table 1). Energetic deficiency throughout the growth rate may result in a disrupted skeletal development, which may also affect the pelvic bone ring. The skeletal growth of cattle is completed at the age of 2–3 years (35), which is in line with our data on body length and other frame measurements (SH, pelvic measurements, and MA), which showed an increase with age (Figure 4). The measurements of our study also agree with other studies (9, 36, 37).

The animals entered puberty throughout the study period, suggesting a progress of body fat metabolism and skeletal growth (9) related to hormonal changes. In the literature, serum P4 values of ≥2 ng/ml are described to detect the onset of puberty (30). In our study, an average 27.8% of all study animals were below 2 ng/ml P4, suggesting a prepubertal stage (farm A: 7 animals, 17.5%; farm B: 8 animals, 20%; farm C: 10 animals, 25%; and farm D: 15 animals, 37.5%). The majority of prepubertal cattle was found in farm D (37.5%) related with enhanced body fat mass and smallest SH.

Thus, we conclude that after puberty, changes occur in fat deposits that promote post-pubertal skeletal growth.

By analyzing serum E2, there was a highly significant negative correlation of E2 with pelvic width and SH, supporting the modulating function of E2 on bone metabolism (38). Furthermore, there is a highly significant positive correlation between E2 and fat content. This might be explained by the fact that (pre-)adipocytes convert androgens into estrogens through P450 aromatase (39). We found the lowest E2 values in combination with the lowest fat content and highest skeletal growth in farm B, which suggests a possible connection between fat development and skeletal growth.

Leptin is another hormone related to puberty and fat metabolism with significant lower levels in early than in late pubertal cattle (40). In our study, there was a strongly significant positive correlation between serum leptin content and pelvic width, explaining its effects on bone growth (41). Furthermore, it is also postulated that total body fat content correlates with serum leptin concentrations (42), which we could not find. However, our data detected a highly significant positive correlation between serum leptin and BM, supporting a more prominent effect of leptin on bovine skeletal growth. We obtained variable serum leptin levels in the heifers from 8 to 15 months of age, which can be explained by the fact that leptin is mainly produced by adipocytes (43) in subcutaneous fat deposits (39), which do develop until the ninth month of life (11).

Previous studies on cattle fat metabolism were limited according to data about BCS, BFT, and the BM (20, 44). Summarizing the different data and our study results, BCS and BFT are not the optimal parameters for the interpretation of body condition in rearing cattle. On the one hand, this might be explained by the fact that using the BCS is just based on subjective determination (10, 12), and the scoring system according to Edmonson et al. (10) is only well-established for adult cattle. The measurement of BFT on the other hand seems to be a more objective method for body condition (20) and correlates well with BCS for adult cattle. In contrast, Abhishek et al. (45) showed in their work that the use of BCS and BFT resulted in lower correlations for primiparous than for multiparous cattle. Similar results were reported for BM and BCS in primipara. They concluded that primiparous animals use nutrients more for growth than development of the subcutaneous depot. However, BM alone also does not allow any statement on body composition (20). In our study, farm B had the highest BM with the highest SH and lowest fat content, whereas farm D had lowest BM with the smallest SH and highest body fat content.

With our “*BIA Heine*” method, it might be possible to evaluate BCS specifically for rearing cattle, based on the fact that the measurement is independent of any fat deposits.

To analyze the different body conditions throughout the variable rearing intensity, the whole-body analysis is recommended in literature as the “gold standard” to obtain the fat content of the cow (12). However, this technique requires slaughtering of the animals, which can be avoided by using our non-invasive “*BIA Heine*” method. Interestingly, both techniques, “*BIA-Heine*” and the BFT measurement, verified by whole-body analysis (11) obtained similar results on the fat content of the animals. While BFT is based on the measurement of the subcutaneous deposits (which develop until the ninth month), “*BIA-Heine*” includes all deposits, so the BIA technique can be used regardless of age. Therefore, the “*BIA Heine*” is an exceptional non-invasive technique for fat content analysis, supported by already published data based on age, breed, and feeding (12, 19).

Our hypothesis supposes the rearrangement of stored fat deposits in heifers. This was confirmed by our data demonstrating a reduction in fat deposits until the age of 15 months. At this time point, the stored energy is primarily used for growth processes (44), supported by our high MA results.

According to IGF-1, which is mainly produced by the liver, but also by the fat tissue, the lipid mobilization is associated with low IGF-1 values (46). IGF-1 can influence the skeletal growth (39) in heifers, which is in agreement with our results from farm B. These animals had the lowest IGF-1 concentration and the lowest fat mass combined with high serum FFA (Figure 5). Farm B heifers additionally displayed the highest frame development, which supports our hypothesis of rearranged stored energy in support of skeletal growth. With regard to the proportion of post-pubertal animals, which was higher in farm B compared to farm D, the lowest frame formation and lowest serum FFA concentrations in farm D animals might be due to the fact that this rearrangement of fat deposits is a post-pubertal process.

Another important point is that IGF-1 concentration can be modulated by genetics (47–49). Low IGF-1 concentrations do correlate with high milk production, suggesting an effect on the somatotropic axis (49). In farms A and D with various genetics and high IGF-1 levels (Figure 5), a higher beef genetic than pure milk genetic is documented. Interestingly, all these animals had also a lower frame than the animals of farms B and C. We additionally found an association between the liver enzyme GLDH and the hormones IGF-1 as well as E2 in farms A and D, which can be explained by the fact that GLDH is bound to the mitochondrial membrane of the hepatocytes (50). This is in agreement with the expected high growth performance of the animals, which is in conjunction with increased liver metabolism (51).

These results support the hypothesis that fat remodeling is IGF-1 dependent, in order to support skeletal growth. In addition, IGF-1 levels correlate significantly with insulin, which is the most important anabolic hormone (41). Furthermore, our study showed significant positive correlations between IGF-1 and BCS, BFT and MA, as well as a significant negative correlation between IGF-1 and the percentage of body fat content. Regarding insulin, there is a significant positive correlation with BFT and BCS, as well as with MA, but no correlation with body fat content.

Based on the results of the present study, we recommend an individualized rearing management for each farm, which reduces post-pubertal adiposity in Holstein heifers, which (based on our data) must occur after 15 months of age. Furthermore, it can be an option to establish a BCS designed for rearing cattle using “*BIA Heine*.” Serum IGF-1 turned out to be an important hormone for the rearrangement of body fat deposits and the support of skeletal growth, which primarily started in the post-pubertal phase. As a final remark, it is necessary to reconsider the current feed compositions for heifers, especially in terms of protein supply and feed quality with special attention to optimal DMI. This will guarantee high growth rates of heifers, which are genetically determined. In conclusion, the establishment of the non-invasive “*BIA Heine*” technique provides an important contribution to monitor fat deposition in growing heifers with regard to improvement in animal health and welfare. We finally found causative differences between the four farms during the rearing period (like genetic differences, various food quality, and, feeding management), which might explain the differences in the body development of the animals and the stillborn rates.

## Data Availability Statement

The raw data supporting the conclusions of this article will be made available by the authors, without undue reservation.

## Ethics Statement

The animal study was reviewed and approved by Landesdirektion Leipzig, Germany, file reference 48/18. Written informed consent was obtained from the owners for the participation of their animals in this study.

## Author Contributions

KH wrote the entire manuscript. Also was KH primarily responsible for analyzing the data collected. VK and MK assisted during the study period and data collection. JG and SR planned the study and analyzed the endocrine parameters. IV performed the feed analysis and reviewed the manuscript. LB reviewed the manuscript. JL as well as AE obtained the financial resources. IS planned the study design. AE reviewed the study, and data measurements. All authors contributed to the revision of the manuscript and read and approved the submitted version.

## Conflict of Interest

The authors declare that the research was conducted in the absence of any commercial or financial relationships that could be construed as a potential conflict of interest.

## Publisher's Note

All claims expressed in this article are solely those of the authors and do not necessarily represent those of their affiliated organizations, or those of the publisher, the editors and the reviewers. Any product that may be evaluated in this article, or claim that may be made by its manufacturer, is not guaranteed or endorsed by the publisher.

## Abbreviations

BCS: Body-Condition-Score
BFT: Back-Fat-Thickness
BHB: ß-hydroxy butyrate
BIA: Biolectrical Impedance Analysis
BM: body mass
BUN: Urea
CP: crude protein in the total mixed ration
DM: dry mater
DMI: dry mater intake
EIA: enzyme-immunoassay
E2: oestradiol
FFA: free fatty acids
GLDH: glutamate dehydrogenase
GGT: gamma-glutamyl transferase
IGF-1: insulin-growth-factor 1
MA: measuring area
MD: distances between the sensor electrodes
ME: metabolized energy in the total mixed ration
peNDF: physical effective NDF
P4: progesterone
RIA: radio-immunoassay
R50: resistance frequency 50 kHz
SH: sacrum high
TMR: total mixed ration.
